# Carbon dioxide narcosis following cesarean section in a patient with severe pulmonary hypertension: A case report

**DOI:** 10.1097/MD.0000000000039857

**Published:** 2024-10-11

**Authors:** Song Lyu, Min Liao

**Affiliations:** a Department of Anesthesiology, The Second Affiliated Hospital of Hainan Medical University, Haikou, China.

**Keywords:** carbon dioxide narcosis, case report, drug interaction, pulmonary hypertension, sufentanil

## Abstract

**Rationale:**

Managing anesthesia in patients with severe pulmonary conditions involves complex considerations, especially when dealing with high baseline CO_2_ levels. We present a case that demonstrates the challenges and complexities of anesthesia and postoperative analgesia in a patient with severe pulmonary hypertension and a history of lung disease exacerbated by the interactions of protein-bound drugs.

**Patient Concerns:**

A 37-year-old woman at 38 weeks of gestation presented with recurrent chest tightness, shortness of breath, and worsening symptoms over a week, which required emergency medical attention.

**Diagnosis:**

The patient was diagnosed with severe pulmonary hypertension, and echocardiography revealed a pulmonary artery pressure of 106 mm Hg upon admission. Postoperative complications included sudden unconsciousness after low dose (2 µg) sufentanil administration, indicative of carbon dioxide narcosis that could compound pharmacological interactions and her underlying condition.

**Interventions:**

The patient underwent a cesarean section under spinal anesthesia, which was complicated postoperatively by respiratory depression, requiring naloxone administration and intensive care.

**Outcomes:**

Despite initial postoperative challenges, the patient’s condition stabilized, allowing eventual discharge.

**Lessons:**

The clinical course highlighted the need for careful monitoring and prompt intervention in anesthesia in patients with severe pulmonary hypertension, particularly when administering multiple protein-bound drugs. Drug interactions can exacerbate the underlying condition, necessitating diligent oversight to prevent severe complications such as carbon dioxide narcosis.

## 1. Introduction

Hypercapnia, characterized by elevated serum CO_2_ levels, can present a wide range of clinical manifestations, with carbon dioxide narcosis being the most severe. A key indicator of carbon dioxide narcosis is a reduced level of consciousness.^[1]^ Prompt recognition and treatment of carbon dioxide narcosis are crucial, as failure to address it can lead to coma or fatal outcomes.

## 2. Case presentation

A 37-year-old woman (65 kg, 158 cm) at 38 weeks of gestation was admitted to the hospital with recurrent chest tightness and shortness of breath for more than 2 years, which had worsened in the past week, and an inability to lie down. Her obstetric history included 1 cesarean section and 3 normal deliveries. Two years earlier, she was hospitalized for edema in both lower extremities that persisted for more than a month postpartum. Echocardiography at that time indicated a pulmonary artery pressure (PAP) of 94 mm Hg (normal 11–20 mm Hg). A CT scan revealed evidence of past tuberculosis. After symptomatic treatment, her PAP was reduced to 36 mm Hg, and she was discharged with improved symptoms.

Upon admission on July 3, 2023, at 38 weeks of gestation, the patient presented with a deterioration of her condition over the past week. Her blood pressure was 144/98 mm Hg, her heart rate was 109 beats per minute, her respiratory rate was 32 breaths per minute, and her oxygen saturation (SpO_2_) was 72%. The patient was coughing and breathing while sitting, exhibiting pitting edema below the umbilicus. A blowing murmur was audible during tricuspid systole, and scattered wet rales were heard in both lungs. Laboratory findings revealed a hemoglobin level of 91 g/L, N-terminal pro-brain natriuretic peptide (NT-proBNP) of 2025.00 pg/mL, albumin of 2.53 g/dL (normal 3.5–5.5 g/dL), and aspartate aminotransferase of 253 units/L (normal 8–33 units/L). An echocardiography showed severe PAP (approximately 106 mm Hg). She was diagnosed with severe pulmonary hypertension combined with pregnancy and was admitted for multidisciplinary treatment. She was transferred to the intensive care unit (ICU) for preoperative preparations.

Two hours before surgery on July 4, 2023, blood gas analysis revealed a pH of 7.17, partial pressure of oxygen (PO_2_) of 76 mm Hg, partial pressure of carbon dioxide (PCO_2_) of 75 mm Hg, hemoglobin of 92 g/L, and N-terminal pro-brain natriuretic peptide of 1188 pg/mL. At 8:00 am, the patient received 4 L/min of oxygen through a face mask, achieving a SpO_2_ of 94%. Puncture and manometry of the left radial artery showed a pH of 7.23, PCO_2_ of 61 mmol/L, and a PO_2_ of 227 mmol/L. Two intravenous cannulas were placed in the internal jugular and femoral veins, establishing 7 intravenous access points for medications, including oxytocin, norepinephrine, dopamine, nitroglycerin, albumin, erythrocytes, and propofol.

Spinal anesthesia was administered in the L2–3 interspace in the sitting position using a 25-gauge needle, with 3 mL of isobaric fluid injected intrathecally (bupivacaine 10 mg, morphine 70 µg, nalbuphine 70 µg). The patient underwent surgery in a semi-sitting position, with a sensory level at T6 (pinprick test at T8). The patient’s vital signs remained stable, and propofol (8 mL/h) induced drowsiness. The obstetrician proceeded cautiously to prevent sudden hemodynamic changes. The procedure lasted 1.5 hours and included a cesarean section with tubal ligation and uterine artery ligation. The fetus had an Apgar score of 10 and was discharged after 10 days.

After delivery, 20 mg of furosemide was immediately administered to the patient. The fluid infusion totaled 180 mL. The blood loss was 500 mL, and the urine output was 300 mL. The patient’s vital signs remained stable during the operation. At the end of the procedure, a bilateral quadratus lumborum block was performed with 0.25% ropivacaine (40 mL).

During preparation for patient-controlled analgesia, the patient suddenly lost consciousness after receiving 2 µg of sufentanil intravenously. Mask-controlled ventilation and 0.2 mg of naloxone were administered. Blood gas analysis showed a pH of 7.1, PCO_2_ of 91 mm Hg, and PO_2_ of 124 mm Hg. The patient regained consciousness 20 minutes later and was transferred to the ICU.

**Figure 1.**
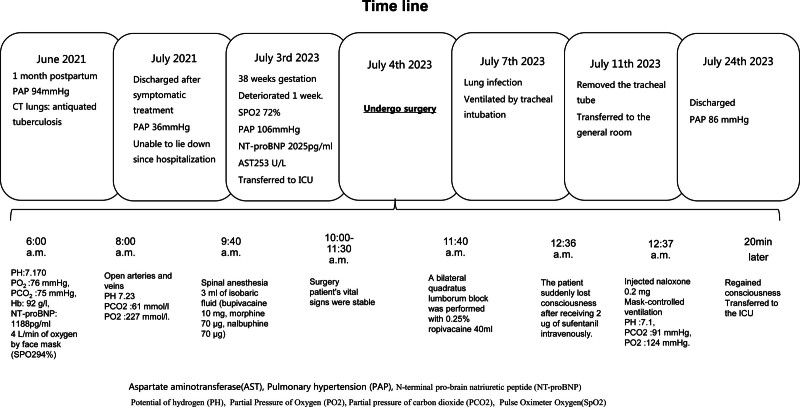
Time line.

On the third postoperative day, the patient developed a lung infection and required tracheal intubation. On the seventh postoperative day, the tracheal tube was removed and the patient was transferred to the general room. She was discharged 21 days later with a PAP of 86 mm Hg. Figure 1 shows the detailed timeline.

## 3. Discussion and conclusions

**Figure 2.**
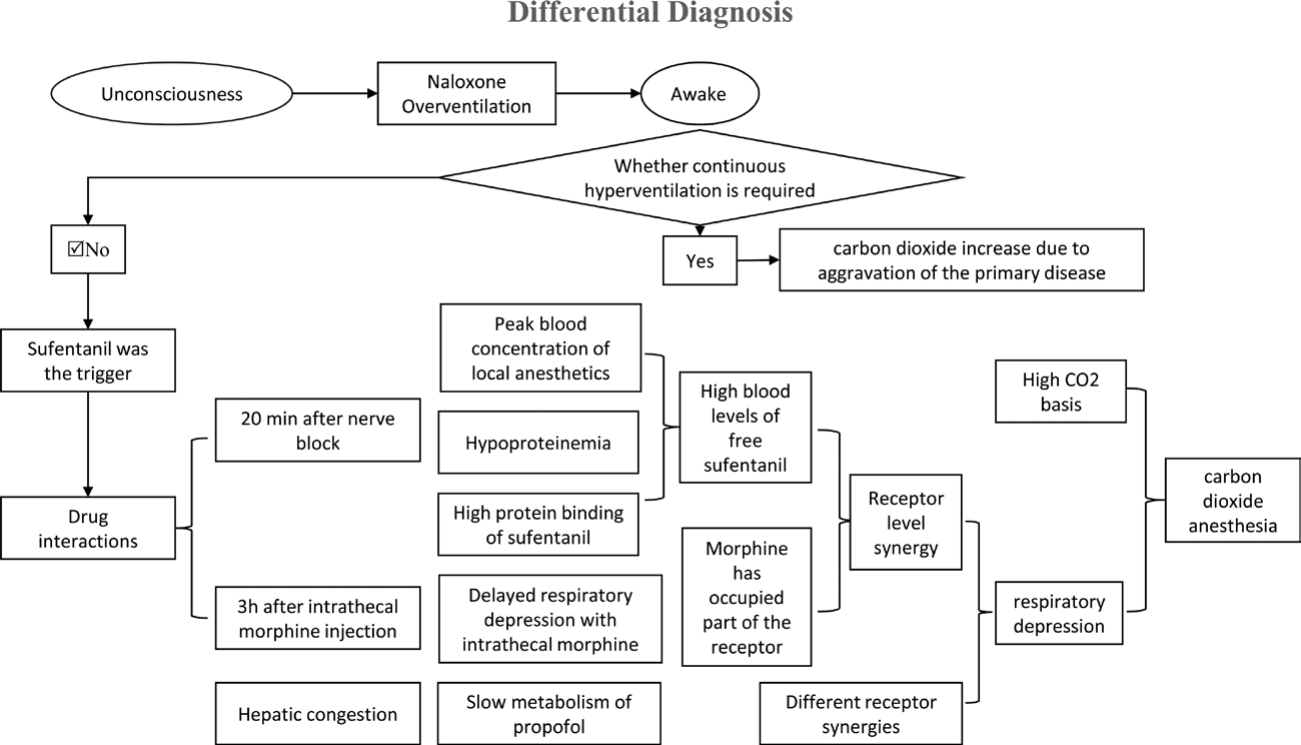
Differential Diagnosis.

The differential diagnosis of the patient’s sudden loss of unconsciousness mainly focused on the interplay between drug interactions and the physiological impacts of the patient’s underlying lung disease. A low dose of sufentanil induced unconsciousness, leading to significant clinical observations. An increase in CO_2_ levels exacerbated her primary lung condition, necessitating the use of naloxone to counteract respiratory depression. After naloxone administration, the patient remained awake without needing continuous mechanical ventilation in the ICU. This suggested that the respiratory collapse in this case was not primarily due to pulmonary pathology. We hypothesize that respiratory collapse resulted from 3 main factors: increased blood drug concentrations, synergistic effects at the opioid μ-receptor level, and combined influence of γ-aminobutyric acid (GABA) and μ receptors (Fig. 2).

Managing anesthesia in patients receiving multiple protein-bound drugs is complex and requires careful consideration of drug interactions and patient-specific factors. Ropivacaine, which is highly protein-bound at 94%, reaches its peak plasma concentration approximately 30 minutes after administration by quadratus lumborum block.^[2,3]^ Sufentanil and morphine, with high protein binding rates, also compete for plasma protein binding sites.^[4,5]^ In this case, prior administration of morphine, despite its lower protein binding affinity, probably occupied some binding sites, reducing their availability for sufentanil and ropivacaine. This problem was exacerbated by the patient’s low albumin levels, which decreased the protein binding capacity, thereby increasing the free fractions of these potent drugs. Consequently, the elevated free fraction of sufentanil led to significant analgesia and increased the risk of opioid-related side effects, including respiratory depression.

Intrathecal administration of 75 µg of morphine is considered safe.^[6]^ Morphine has been applied to anesthesia in women with pulmonary hypertension^[7]^ without affecting minimal ventilation and response to CO_2_.^[8]^ However, this patient experienced respiratory depression 3 hours after administration, which is consistent with documented cases of hypercapnia^[9]^ and delayed respiratory depression associated with intrathecal morphine.^[10]^ Subsequent intravenous administration of sufentanil may have amplified this effect,^[11]^ suggesting a possible synergistic impact at the opioid μ-receptor level, which likely exacerbated respiratory depression.

Propofol, a GABA receptor agonist with a favorable pharmacokinetic and pharmacodynamic profile, has been the most widely used intravenous anesthetic for the past 3 decades.^[12]^ Primarily metabolized in the liver to inactive metabolites and excreted by the kidneys, the metabolism of propofol can be altered in patients with liver dysfunction, leading to prolonged drug exposure and accumulation. Although propofol was discontinued 1 hour earlier, the patient’s impaired liver function probably affected drug metabolism,^[13]^ making it impossible to exclude possible synergistic effects between propofol and sufentanil at the GABA receptor level, further complicating the respiratory situation. These pharmacokinetic and pharmacodynamic changes, coupled with the patient’s underlying high baseline CO_2_ levels due to lung disease, could have led to carbon dioxide narcosis in this case.

This case demonstrates the complexity of anesthesia management in patients with lung disease, highlighting the need for careful monitoring and consideration of drug interactions to prevent serious complications such as carbon dioxide anesthesia. Management considerations included continuous hyperventilation to manage elevated CO_2_ levels effectively.

## Author contributions

**Conceptualization:** Song Lyu.

**Data curation:** Song Lyu, Min Liao.

**Formal analysis:** Song Lyu.

**Funding acquisition:** Song Lyu.

**Investigation:** Song Lyu.

**Writing – original draft:** Song Lyu, Min Liao.

**Writing – review & editing:** Song Lyu.
